# The cortical sensory representation of genitalia in women and men: a systematic review

**DOI:** 10.3402/snp.v5.26428

**Published:** 2015-03-10

**Authors:** Fadwa Cazala, Nicolas Vienney, Serge Stoléru

**Affiliations:** INSERM U669, Université Paris Descartes, Paris, France

**Keywords:** genitalia, penis, clitoris, primary somatosensory cortex, secondary somatosensory cortex, homunculus, insula, somatosensory representation, somatosensory organization, sexual arousal

## Abstract

**Background:**

Although genital sensations are an essential aspect of sexual behavior, the cortical somatosensory representation of genitalia in women and men remain poorly known and contradictory results have been reported.

**Objective:**

To conduct a systematic review of studies based on electrophysiological and functional neuroimaging studies, with the aim to identify insights brought by modern methods since the early descriptions of the sensory homunculus in the primary somatosensory cortex (SI).

**Results:**

The review supports the interpretation that there are two distinct representations of genital sensations in SI, one on the medial surface and the other on the lateral surface. In addition, the review suggests that the secondary somatosensory cortex and the posterior insula support a representation of the affective aspects of genital sensation.

**Conclusion:**

In view of the erogenous character of sensations originating in the genitalia, future studies on this topic should systematically assess qualitatively as well as quantitatively the sexually stimulating and/or sexually pleasurable characteristics of sensations felt by subjects in response to experimental stimuli.

Since the publication of the well-known somatosensory homunculus (Penfield & Boldrey, 1937; Penfield & Rasmussen, 1950) which represents the functional somatotopic organization of the primary sensory cortex, numerous studies have added new information to what we know about the somatosensory cortical representation of the human body. In this paper, we focus on the cortical representation of the human genitalia. Genitalia are defined here as the clitoris, labia majora, labia minora, and the opening of the vagina (introitus) in females, and as the penis and the scrotum in males. As noted by Kell, von Kriegstein, Rosler, Kleinschmidt, and Laufs (2005), the drawings by Penfield and Rasmussen (1950) in essence confirmed older charts but displaced them in popularity because, at a single glance, they compellingly illustrated somatotopy as a key principle in the layout of these cortices. However, on closer inspection of the somatosensory homunculus, one inevitably notes a violation of somatotopic continuity regarding the localization of the genitalia. Why should they be represented below the toes in the mesial wall?

In the study by Penfield and Rasmussen (1950), which relied on electrical stimulation during open brain surgery, there is a lack of data about the cortical somatosensory representation of genitalia. Genital sensation was reported by only three of 400 patients in response to electrical stimulation of the cortex adjacent to the central fissure, 1 cm posterior to the representation of the upper leg and lower trunk. Moreover, in this study no genital sensation was reported in response to the stimulation of the mesial surface of the postcentral gyrus, a region supposed to be involved in the representation of the genitalia (Foerster, 1936; Pfeifer, 1920). However, this negative finding was not incorporated into the drawing of the sensory homunculus. ‘Presumably rectum and genitalia should be placed above feet, that is within the longitudinal fissure, but our evidence is not sufficient for conclusion and they seem to be somewhat posterior to feet’, wrote Penfield and Boldrey (1937). As noted by Michels, Mehnert, Boy, Schurch, and Kollias (2010), the somatotopic representation of the genital region was especially hard to assess due to difficulties to answer related to a sense of shame.

Since these early studies, several experiments using modern neuroscientific methods have been conducted, with diverging results. Essentially, some studies concluded that the somatosensory representation of genitalia lay on the uppermost part of the somatosensory cortex (e.g. Georgiadis et al., 2006; Kell et al., 2005), while others found it was located in the mesial wall of the hemisphere, i.e. in the paracentral lobule (e.g. Allison, McCarthy, Luby, Puce, & Spencer, 1996; Mäkelä et al., 2003; Nakagawa et al., 1998). Here, we review the literature on the somatosensory organization of the genitalia in women and men, with an aim to understand the conflicting results obtained thus far.

In addition, instead of focusing only on the representation of the genitalia in the primary somatosensory cortex (SI), we also review evidence related to the genital somatosensory representation in the secondary somatosensory cortex (SII) and in the insula, topics that have been rarely documented. This additional focus stems from the specific character of sensations coming from genitalia. Many previous studies have focused on fine touch (epicritic sensation), rather than on crude touch and pleasurable sensations. Contrasting with these studies, the present work tries to identify the cortical representation of the various modalities of sensations from the genitalia. One strong reason to focus on SII is that, surprisingly, none of the various meta-analyses of neuroimaging studies of brain responses to sexual stimuli reported activation in SI, even those that focused on the neural correlates of erection; conversely, these meta-analyses agreed remarkably on demonstrating an activation in SII (Kühn & Gallinat, 2011; Poeppl, Langguth, Laird, & Eickhoff, 2014; Sescousse, Caldú, Segura, & Dreher, 2013; Stoléru, Fonteille, Cornélis, Joyal, & Moulier, 2012).

Defining the somatotopic sensory organization of the genitalia is not only important to fill a gap in the drawing of the sensory homunculus and resolve a controversy about it, but also because this organization is the basis of the strongest erotic sensations. In a survey based on an online Erogenous Zone Questionnaire and a scale on which 793 participants rated, in terms of level of arousal, the ability of 41 different body areas to facilitate sexual arousal (with 0: ‘no erotogenic stimulation’, and 10: ‘highest stimulatory capacity’), the body parts with the highest scores were the clitoris (mean=9.17; SD=2.12) and the penis (mean=9.00; SD=2.50) (Turnbull, Lovett, Chaldecott, & Lucas, 2014).

## Methods

We conducted a systematic review of research reports, based on PubMed, Scopus and Google Scholar databases, with, on the one hand, the terms ‘somatosensory cortex’, ‘homunculus’, ‘somatosensory representation’, ‘somatosensory organization’, ‘insula’ crossed with, on the other hand, ‘genitals’, ‘genitalia’, ‘penis’, ‘testis’, ‘scrotum’, ‘clitoris’, ‘vagina’, ‘vulva’, ‘labia’, ‘sexual arousal’, ‘erection’. Further studies were found by tracing the references cited by identified original papers and review articles, and by identifying papers that cited the identified papers and review articles. Each article was carefully read to make sure it fulfilled all the following criteria: (1) sampled human participants (women and/or men); (2) reported explicitly the cortical location of the representation of genitalia, i.e. specified tridimensional coordinates and/or an anatomical label; (3) used a brain imaging technique [magnetic resonance imaging (MRI), functional MRI (fMRI), positron emission tomography (PET), electroencephalography (EEG), electrocorticography (ECoG), magnetoencephalography (MEG)], evoked potentials, or a direct brain stimulation technique; and (4) used methods based on (i) a stimulation of genitalia [direct stimulation (tactile or electrical) or indirect stimulation (erotic stimulation leading to sexual arousal)] or (ii) an electrical stimulation of the somatosensory cortex, or (iii) brain lesions, with possible associated seizures.

## Results

Following the above-mentioned criteria, 23 articles were found to be eligible, including healthy participants (*n*=19) or patients (*n*=2) or both patients and healthy participants (*n*=2). Seven articles studied a female sample, 14 studied a male sample, while two studied a mixed male/female sample. In addition, our search identified four meta-analyses of functional neuroimaging studies of sexual arousal that were relevant to our focus.

Results are presented in Table 1. These 23 articles are presented below in two separate sections according to the temporal resolution of the imaging technique employed: (i) studies based on techniques with higher temporal resolution, i.e. MEG, EEG or somatosensory evoked potentials (SEPs) (seven studies), and (ii) studies that used fMRI or PET (16 studies), characterized by lower temporal resolution. This dichotomy was used because a preliminary examination of results indicated that these categories of techniques might lead to different results.

**Table 1:** Methods and results of studies of the localization of the somatosensory representation of genitalia

Authors	Year	Sample	Condition	Method for localization	Stimulation method	Induced sexual arousal	Somatotopic sensory organization of genitalia [*x*, *y*, *z* coordinates]		

							Primary sensory cortex	Secondary sensory cortex	Insula
I. Original studies									
Allison et al.	1996	29 M/18 W	Epilepsy	EEG	Electrical stimulation of dorsal pudendal nerve	N	Mesial wall of postcentral gyrus, anterior to the foot sensory area	Not investigated	Not investigated
Arnow et al.	2002	14 M	Healthy	fMRI	Erotic videos	Y	No reported activation	[−34, 5, 18]*M	[42, 6, −2]; [38, −10, −4]; [40, −8, −12]*M
Arnow et al.	2009	20/16 F	Healthy/HSDD	fMRI	Erotic videos	Y	No reported activation	Deactivation in healthy: [40, −18, 18]; activation in patients: [−31, 19, 9]*T	No activation
Bocher et al.	2001	10 M	Healthy	PET	Erotic videos	Y	No reported activation	[−54, −25, 21]*T	No reported activation
Bradley et al.	1998	6 M	Epilepsy	Subdural contact electrodes	Electrical stimulation of DPN	N	Postcentral gyrus, lateral to midline + mesial wall	Not investigated	Not investigated
Brunetti et al.	2008	18 M	Healthy	fMRI	Erotic videos	Y	No reported activation	[49, −21, 21], [−52, −23, 20]*T	[40, −1, 4], [−41, 0, 5]*T
Ferretti et al.	2005	10 M	Healthy	fMRI	Erotic videos	Y	No reported activation	[50, −22, 20], [−52, −20, 17]*T	[38, −5, 8], [−39, −3, 12], [−40, −7, 10]*T
Georgiadis et al.	2006	12 W	Healthy	PET	Manual stimulation of the clitoris by partner	Y	Postcentral gyrus, lateral to midline Arousal before orgasm: [16, −40, 68]; [−20; −44, 66]. Orgasm: [16, −36, 66]*M	Inferior parietal lobule [−52, −28, 40]*M	[36, −18, 14]*M
Georgiadis et al.	2009	11 M/12 F	Healthy	PET	Manual genital stimulation by partner	Y	Common activations for M and W: [−20, −38, 64]*M	Common activations for M and W: [−54, −22, 28]*M	Activation stronger in W: [40, −20, 14]*M
Georgiadis et al.	2010	16 M	Healthy	Perfusion fMRI	Manual penile stimulation by partner	Y	Paracentral lobule [8, −38, 72]*M correlation with circumference Postcentral gyrus, lateral to midline [20, −42, 68]*M: correlation with circumference variations	[50, −32, 16]*M [48, −38, 16]*M [−48, −38, 26]*M [50, −22, 22]*M	[32, 24, 8], [32, 28, −2], [32, 22, 2], [32, 16, −6], [−36, 6, 0], [−34, 30, −4], [−38, 2, −10]*M
Guérit and Opsomer	1991	5 M/5 W	Healthy	EEG	Electrical stimulation of DPN and DCN	N	Surmised location: Mesial wall of postcentral gyrus	No reported activation	No reported activation
Haldeman et al.	1983	5 W	Healthy	EEG	Electrical stimulation of the perineal nerve	N	Surmised location: Mesial wall of postcentral gyrus	No reported activation	No reported activation
Hu et al.	2008	20 M	Healthy	fMRI	Erotic film	Y	Lateral postcentral gyrus [32, −36, 66]*M	No reported activation	[−38, 0, 6], [−40, 0, −4], [38, 6, −10]*M
Kell et al.	2005	8 M	Healthy	fMRI	Tactile stimulation of the penis with toothbrush	N	Lateral postcentral gyrus [24, −33, 72]*M	Prepuce: [61, −21, 14]; [−63, −15, 15]; [53, 0, 0]; [−55, 6, 2]; penile shaft: [61, −21, 16];[−63, −22, 34]; [63, −11, 12]; [−51, −4, 4]*T	No reported activation
Komisaruk et al.	2011	11 W	Healthy	fMRI	Stimulation of clitoris (with hand), vagina and cervix (with stimulator)	Likely	Mesial wall of postcentral gyrus [no report of coordinates]	Activated [no report of coordinates]	No reported activation
Mäkelä et al.	2003	7 M	Healthy	MEG	Stimulation of DPN	N	Mesial wall of postcentral gyrus [−4, −34, 54; 3, −33, 54]*T	[53, −14, 23]; [51, −15, 17]; [−43, −20, 14]; [−47, −23, 19]*T	No reported activation
Michels et al.	2010	15 W	Healthy	fMRI	Electrical stimulation of DCN	Intermediary	Postcentral gyrus, lateral to midline [−18, −38, 63; 19, −37, 57]*T	[56, −2, 12]; [57, −22, 29]; [−53, −2, 10]; [−55, −17, 21]; [−57, −27, 26]*T	[−31, 3, 16], [−48, −37, 20], [41 1 12]*T
Moulier et al.	2006	10 M	Healthy	fMRI	Erotic photographs	Y	Postcentral gyrus, lateral to midline [39, −46, 65] Paracentral lobule [0, −27, 54; −9, −42, 69; 9,−39, 72]*M	[−54, −33, 27]*M	[−36, 18, 0], [−42, −15, −3], [−45, −3, 3], [39, 3, −9], [45, 15, −6], [−45, −3, 6], [36, 18, 9]*M
Mouras et al.	2008	8 M	Healthy	fMRI	Erotic videos	Y	Paracentral lobule [−6, −30, 57]*M	[−54, −18, 21]; [60, −12, 21]*M	[−42, 3, 6], [−33, 18, 0], [−39, 12, −9], [42, 6, −6], [−42, −6, −6], [42, 0, 9]*M
Nakagawa et al.	1998	5 M	Healthy	MEG + sMRI	Electrical stimulation of DPN	N	Mesial wall: medial end of central sulcus	No reported activation	No reported activation
Pukall et al.	2005	28 W	Healthy/vulvar vestibulitis syndrome	fMRI	Non-painful pressure to the posterior vulva	N	No reported activation	[−54, −22, 22], healthy; [−58, −24, 20], patients*T	[−38, −10, 16] healthy; [−34, −2, 14] patients*T
Redouté et al.	2000	9 M	Healthy	PET	Erotic photographs and videos	Y	Postcentral gyrus, lateral to midline [−16, −26, 72]*M	[−72, −24, 22]*M	No reported activation
Yang and Kromm	2004	77 W	Healthy	EEG	Electrical stimulation of DCN and of vagina	N	Surmised location: Mesial wall of postcentral gyrus	No reported activation	Not investigated
II. Meta-analyses									
Kuhn and Gallinat	2011	Meta-analysis 154 M	Healthy	fMRI, PET	Erotic photographs and videos	Y	No reported activation	[54, −31, 31];[−53, −23, 21]*M	[45, 6, −3]; [−42 −2, 6]; [−34, 16, −1]^*M^
Poeppl et al.	2014	Meta-analysis 227 M	Healthy	fMRI, PET	Erotic photographs and videos	Y	No reported activation	[56, −20, 20]; [−54, −22, 18]*M	[44, −2, 4]; [46, 12, −6]; [−44, −2, 6]; [−34, 18, 0]*M
Sescousse et al.	2013	Meta-analysis 443 (M + F)	Healthy	fMRI, PET	Erotic photographs and videos	Y	No reported activation	[56, −26, 38]*M	[−38, 14, −12]; [34, 10, −6]*M
Stoléru et al.	2012	Meta-analysis 235 M	Healthy	fMRI, PET	Erotic photographs and videos	Y	No reported activation	[50, −24, 22]; [−52, −28, 20]*M	[40, 6, −6]; [36, 18, −14]; [40, −2, 6]; [−42, 0, 6]; [−40, 12, −10]*M

DCN, dorsal clitoral nerve; DPN, dorsal penile nerve; EEG, electroencephalography; F, females; fMRI, functional magnetic resonance imaging; HSDD, hypoactive sexual desire disorder; M, males; MEG, magnetoencephalography; N, No; PET, positron emission tomography; sMRI, structural magnetic resonance imaging; Y, Yes.

*M MNI coordinates

*T Talairach coordinates

### Studies based on EEG, MEG, and SEPs

In all studies based on EEG, MEG, or SEPs, electrical stimulation of the dorsal clitoral nerve (DCN), the dorsal penile nerve (DPN), or of genitalia was applied while recording and mapping SEPs or somatosensory evoked magnetic fields (SEFs).

Nakagawa et al. (1998) studied a sample of five healthy men in whom they stimulated the DPN while recording brain responses with MEG. Data obtained by MEG were combined to structural MRI data. The source locations of all SEFs were estimated using a current dipole model in the best-fit sphere. It was found that all SEF sources were localized in the medial wall of the somatosensory cortex, i.e. in the central sulcus contralateral to the stimuli.

Using MEG, Mäkelä et al. (2003) recorded SEFs to DPN, posterior tibial nerve (PTN), and median nerve stimulation in seven healthy men. While SEFs peak latencies of DPN and PTN did not differ significantly, DPN elicited in all participants a response in the mesial part of the somatosensory cortex.

Guérit and Opsomer (1991) recorded SEPs in a mixed sample of five healthy males and five healthy females. This was done during the electrical stimulation of the DPN, DCN, and the bilateral PTN. While they showed that PTN and DPN/DCN SEPs differed in latency (DPN/DCN>PTN), authors recorded a response in central and parietal midline during DPN/DCN stimulation. They surmised that both the smaller amplitudes of P38 and P56 and the weaker gradients obtained after DPN/DCN stimulation compared with PTN stimulation could be explained by the deeper localization of the somatosensory DPN/DCN receiving area in the interhemispheric fissure. In addition, they proposed that the dependence of DPN/DCN SEPs on smaller sensory fibers could account for their longer latency compared with PTN SEPs.

In line with Guérit and Opsomer (1991), Allison et al. (1996) studied a mixed sample (29 men, 18 women) but included only epileptic patients. SEPs were recorded intraoperatively following two methods. Sixteen patients were involved in stimulation of PTN, dorsal pudendal, median, and trigeminal nerves, while recording SEPs. Others (*n*=31) were studied after a chronic cortical implantation of electrode strips. Based on the first group, Allison et al. (1996) showed that genital sensations were represented in the mesial wall of the somatosensory cortex, about 17 mm anterior to the foot sensory area, close to the cingulate sulcus. They suggested that the proximity of cortical stimulation points eliciting genital sensations and of SEPs to the cingulate sulcus indicated that the genital representation resided mainly within the cingulate sulcus. According to this suggestion, this localization might explain the rarity of genital sensations in response to stimulation of the surface of the mesial wall. In their study, cortical stimulation rarely induced sensations in the genital area.

Using EEG, Haldeman, Bradley, Bhatia, and Johnson (1983) and Yang and Kromm (2004) studied exclusively female genital somatosensory representation. While Haldeman et al. (1983) studied a small sample of five healthy women, Yang and Kromm (2004) studied 77 healthy participants. Yang and Kromm (2004) used EEG to record SEPs during the stimulation of the dorsal nerve of the clitoris and the perineal nerve. While Haldeman et al. (1983) recorded responses from ‘2 cm behind the Cz electroencephalographic recording site’, in Yang and Kromm (2004) study the active electrode was positioned at Cpz, ‘which overlies the sensory cortex and is a midline recording site overlying the central sulcus, between the two cerebral hemispheres’. Based on previous studies, Haldeman et al. (1983) and Yang and Kromm (2004) surmised that recorded SEPs originated from the medial wall of the postcentral gyrus.

Bradley, Farrell, and Ojemann (1998) studied a sample of six epileptic male patients refractory to drug therapy. Participants underwent a subdural placement of contact grid electrodes before any brain resection. DPN stimulation led to evoked cortical potentials beginning approximately 3 cm lateral to the interhemispheric fissure and extending down into the interhemispheric fissure another 3 cm.

In summary, most of the studies based on EEG, MEG or on recorded SEPs agreed with the representation of the homunculus proposed by Penfield and Rasmussen (1950). These studies either recorded a response in the medial wall of the somatosensory cortex, with no difference identified between men and women, or surmised that the recorded signals came from that area. However, some of these imaging techniques were limited by their low spatial resolution or by their inability to record events occurring deep in the interhemispheric fissure and results depend on scalp placement of electrodes, sometimes based on a priori assumptions. To avoid some of these pitfalls, one can get further and complementary insights from studies based on other brain imaging techniques such as PET and fMRI.

### Studies based on PET and fMRI

Studies based on PET and fMRI used two very different kinds of genital stimulation: either a direct, tactile, stimulation (Georgiadis & Holstege, 2005; Georgiadis, Reinders, Paans, Renken, & Kortekaas, 2009; Georgiadis et al., 2010; Kell et al., 2005) or an indirect stimulation, mediated by the presentation of sexually arousing visual stimuli (Moulier et al., 2006; Mouras et al., 2008; Redouté et al., 2000). Regarding the direct kind of stimulation, a further distinction should be made between (1) tactile stimulation not intended to be sexually arousing, e.g. stimulation with a toothbrush (Kell et al., 2005) or electrical DCN stimulation (e.g. Michels et al., 2010) and (2) stimulation intended to be sexually arousing (all studies based on visual sexual stimuli and all studies based on masturbatory tactile stimulation).

Michels et al. (2010) used fMRI to map the somatotopic representation of the human clitoris in 15 participants. The experiment consisted in bilateral electrical stimulation of DCN and right hallux (control condition for eight subjects) using a block design. The random-effects group analysis revealed a bilateral activation during clitoral stimulation in the dorsal postcentral gyrus, laterally to the hallux representation. Surprisingly, Michels et al. (2010) found stronger activations in the left somatosensory cortex for bilateral clitoral stimulation. The insula also showed predominantly left-hemispheric activations. Michels et al. suggested that this activation could be explained by the major role played by the insula in viscerosensory processing (e.g. Craig, 2003), and therefore, this activation fits well with the rather specialized, almost viscera-like, innervation of the clitoris.

Kell et al. (2005) conducted an fMRI study on eight healthy male subjects. Using a block design, they stimulated with a toothbrush the left side of the proximal and distal portions of the penis, the left hallux and left lower abdominal wall of participants. Based on ROI analysis, results showed that the hallux was represented in the medial edge of the contralateral postcentral gyrus, while the penile representation was located lateral to the hallux. In other words, the penis was not represented in the mesial wall but on the lateral part of the somatosensory cortex. According to Kell et al. (2005), the previously reported mesial wall activation could be explained by methodological differences. Kell et al. (2005) used tactile stimulation while MEG and EEG studies used artificial electrical stimulation.

Georgiadis et al. (2006) used PET to investigate the brain correlates of orgasm in response to clitoris stimulation in 12 heterosexual healthy women. PET acquisitions were performed in four conditions: (1) rest; (2) stimulation of clitoris by partner (without body movement); (3) orgasm induced by clitoral stimulation, which entailed body movements; (4) stimulation of clitoris by participant’ partner coupled with voluntary rhythmic contractions of the hips, buttocks, abdominal and pelvic floor to control for body movements. The brain somatosensory representation of the clitoris was inferred based on the second condition contrasted with the first one. Results showed that clitoral stimulation led to a bilateral activation of the dorsal surface of postcentral gyrus, with no somatosensory representation of the human genitalia in the paracentral lobule, contrary to the proposed localization by Penfield and Rasmussen (1950). In addition, during orgasm, there was a large cluster surrounding the dorsal central sulcus, centered on the dorsal convexity of SI. Activation in the left inferior parietal lobule, which was the area associated with the highest *Z*-value when the second condition was contrasted with the first one, was interpreted as the activation of the SII. This same area was also more activated in the Stimulation (Condition 2) minus Orgasm (Condition 3) contrast.

In contrast to other fMRI and PET studies, findings by Komisaruk et al. (2011) agreed with those of MEG and EEG studies. They conducted an fMRI study on 11 women to map the somatosensory cortical representation of the clitoris, the anterior wall of the vagina, the cervix and the nipple. While stimulation of the clitoris and of the nipple was done using their right hand, patients used a rounded-top cylinder stimulator for vaginal and cervical stimulation. Stimulation of the left thumb, the left hallux, and the left nipple were performed as control conditions. Thumb and hallux were stimulated by experimenter. Results revealed distinctive brain foci of activation for clitoris, vagina and cervix. The responses to clitoral, vaginal, and cervical self-stimulation were all located in the paracentral lobule, with a superior-to-inferior disposition (clitoris>cervix>vagina). The differential sensory innervation of these parts of the genitalia could explain this result. Moreover, stimulation of the nipple also activated the paracentral lobule. This is in agreement with women's reports that nipple or breast stimulation is erotogenic. Thus, these results seem consistent with Penfield and Rasmussen's homunculus. Komisaruk et al. (2011) interpreted the activation found by other studies in the uppermost part of the somatosensory cortex, just lateral to supero-medial margin of the hemisphere, as a probable consequence of unavoidable mechanical stimulation of the groin in the course of self-stimulation of the cervix. However, close inspection of coronal slices displaying the clusters reported as corresponding to the clitoris, to the vagina and to the cervix suggests that these clusters might be located in the motor part of the paracentral lobule rather than in its sensory part. However, as the coordinates of the activations and of the presented slices are not provided, it is impossible to assess the precise location of activations.

Georgiadis et al. (2010) conducted a perfusion fMRI study on 16 healthy men. The experiment consisted in a phase of penile stimulation by partner until the participant reached the maximum arousal for the first stimulation period, while the second stimulation period ended when ejaculation occurred. This perfusion fMRI session was coupled with a penile tumescence measure done with an ‘erectometer’. The lateral postcentral gyrus was activated by penile stimulation as compared to the pre-stimulation phase (i.e. flaccid penis, <10% of maximum penile circumference) and to the post-stimulation phase (Post-stimulation, i.e. >10% of maximum penile circumference). In addition, while the penile circumference during the onset of penile erection was correlated with rCBF in the right mesial SI, the variations of penile circumference, i.e. the temporal derivative, was correlated with the rCBF in the right lateral SI. SII was reported as activated by penile stimulation, both when compared with the flaccid state and when compared with the post-stimulation phase. However, not all the clusters of activation seem to fall in the classic location of SII, e.g. *x*=50, *y=*−32, *z*=16 (MNI coordinates).

Studies reviewed above mapped somatosensory genital representation during tactile or electrical stimulation of genitalia, or in response to electrical stimulation of the cortex. We now review studies of the somatosensory correlates of visually-induced penile erection (Hu et al., 2008; Moulier et al., 2006; Mouras et al., 2008; Redouté et al., 2000). In Moulier et al. (2006) study, in addition to a cluster lying deep in the interhemispheric fissure, two clusters on the lateral surface of the somatosensory cortices covaried positively with the penile plethysmographic signal. In line with Moulier et al. (2006), Mouras et al. (2008) revealed a positive correlation between the penile response and the BOLD signal in the paracentral lobules. They also reported an activation of the uppermost part of the right lateral somatosensory cortex. However the correlation with penile response was only significant at an uncorrected threshold (*p<*0.001, uncorrected). Similarly, Redouté et al. (2000) reported that the activation in the uppermost part of postcentral gyrus (Brodmann area 3) covaried both with the perception of tumescence and with the objective measure of penile circumference.

Hu et al. (2008) conducted an fMRI study where 10 heterosexual and 10 homosexual male participants were exposed to erotic films. As found by other studies using visual sexual stimuli (Moulier et al., 2006; Mouras et al., 2008; Redouté et al., 2000), Hu et al. (2008) observed an activation of lateral postcentral gyrus in both studied groups. Thus, most of fMRI or PET studies have found that penile erection is related to an activation in the lateral postcentral gyrus, interpreted as the somatosensory representation of men's genitalia.

In summary, while most of the studies based on EEG, MEG or the recording of SEPs agree with the representation of genitalia in the Penfield and Rasmussen homunculus (1950), most of fMRI and PET studies report an activation in the lateral postcentral gyrus, with or without additional activation in the medial part of the somatosensory cortex. Regarding the activation of SII, as shown in Table 1, coordinates of activation corresponded rather closely to the most likely functional localization of SII on the left hemisphere (*x*=−52, *y*=−23, *z*=+18) and on the right hemisphere (*x=*54, *y*=−22, *z*=+20; MNI coordinates) (Eickhoff, Amunts, Mohlberg, & Zilles, 2006). As suggested by Georgiadis et al. (2006), perhaps SII assigns a conscious label to the salience of a somatosensory stimulus, e.g. in the present study, to the perception of the genital stimulation as ‘sexual’.

Finally, regarding the insula, while studies using sexual stimuli tended to find insular activation, most of those relying on neutral stimuli did not report such activation.

## Discussion

### Two distinct representations of genitalia in the SI

Regarding the representation of genitalia in SI, studies reviewed above have reported two distinct representations of genital sensations, one on the mesial surface and the other on the lateral surface of SI. Thus, either one of the localizations is an artifactual finding, possibly related to methodological problems, or there are actually two coexisting cortical representations of genitalia. In the latter case, discrepant findings could be equally valid but would result from different methodologies. Evidence for the coexistence of the two representations stems from studies which found both localizations (Bradley et al., 1998; Georgiadis et al., 2010; Moulier et al., 2006).

One possible interpretation of the coexisting distinct localizations could be that widely different sensations are evoked across studies. For instance, Kell et al. (2005) used a toothbrush to stimulate the left aspect of the penile shaft and the left prepuce (glans in subject 5) in craniocaudal direction at approximately 2 Hz. No sexual responses or feelings were elicited. In this study, superficial skin receptors are likely to have been stimulated. Conversely, in studies where visual sexual stimulation caused erection, sensations were felt as sexually pleasurable and experienced as coming not only from the skin but also from deeper parts of the penis.

### The pleasurable character of genital sensations

The starting point of a discussion of the sensory organization of the genitalia should be a phenomenological analysis of genital sensations. By ‘phenomenology of genital sensations’, we refer to the various characteristics of conscious sensations derived from genital stimulation as experienced from the first-person point of view. There are surprisingly few studies on the phenomenology of genital sensations, either in women or men. Obviously, despite the scanty evidence available, genital sensations are first and foremost pleasure sensations rather that fine touch sensations. These two kinds of sensations should be clearly distinguished because there is little in common, for instance, between a sexually pleasurable penile sensation and a fine touch sensation enabling a subject to determine the shape of a figure drawn on his penile skin. Whereas the first kind of sensation is affective, the second is cognitive. Similarly, regarding clitoral sensations, as seems clear from the relatively scarce literature on the subject, the clitoral sensations are quasi exclusively hedonic (Van de Velde, 1928). As put by Waskul, Vannini, and Wiesen (2007), ‘The clitoris is the most sensitive female sex organ; pleasure is its only known function’. Actually, there seems to be more references in the scientific literature to pain coming from female genitalia than to pleasurable sensations. In women, the neglect of scientific attention to the phenomenology of genital sensations is further complicated by the fact that both the vagina and the clitoris are erogenous zones (Turnbull, Lovett, Chaldecott, & Lucas, 2014). The scientific neglect of the phenomenology of clitoral sensations is reminiscent of the societal silence regarding the role of the clitoris, a silence that has been compared to a symbolic clitoridectomy: ‘societal silence regarding the role of the clitoris may act similarly as a symbolic clitoridectomy’ (Ogletree & Ginsburg, 2000). In a scathing article about the lack of recognition of women's sexual pleasure and of the role of the clitoris in sexual pleasure, Scully and Bart (1973, p. 1049) wrote: “In the last two decades at least one-half of the texts that indexed the topics stated that the male sex drive was stronger than the female's; she was interested in sex for procreation more than for recreation. In addition, they said most women were ‘frigid’ and that the vaginal orgasm was the ‘mature’ response. Gynecologists, our society's official experts on women, think of themselves as the woman's friend. With friends like that, who needs enemies?”

### The different kinds of tactile receptors in genitalia

The two kinds of sensations described above are supported by different kinds of sensory receptors. While encapsulated receptors (Krause-Finger corpuscles) have been found in the human glans (glans penis and glans clitoris), the majority of afferent terminals are represented by free nerve endings. In the human glans penis, the ratio of free nerve endings to corpuscular receptors is approximately 10:1 (Halata & Munger, 1986). Receptors in the female genitalia have been reviewed by Hoyt (2006). In women, the skin of external genitalia is richly provided with touch and pressure receptors. Structurally, these can be classified as free nerve endings, endings associated with the hair follicles, endings associated with specialized epithelial cells (Merkel disks), and encapsulated endings. The density of innervation is especially high on the dorsal surface of the glans of the clitoris. There are almost no organized tactile receptors in the vestibule and clitoral body. Encapsulated mechanoreceptors have been found in the erectile tissue of the clitoris. In the region of the introitus, the vagina is supplied with somatic sensory innervation that conveys information from intraepithelial free nerve endings and lamellated tactile endings in the submucosa. Above the hymen, the vagina and cervix receive visceral sensory fibers. While numerous free nerve endings and occasional lamellar corpuscles occur in the endocervix, the vaginal portion of the cervix is supplied with a smaller complement of free nerve endings. Although the vagina has been proposed as the site of the G-spot, alleged to be a highly erogenous zone located roughly a third of the way up the anterior vaginal wall, it is generally agreed that intraepithelial nerve terminals are rare in the vagina and that lamellated tactile endings are absent, except near the introitus. This appears to rule out the existence of the G-spot. However, there is now good evidence for a rich plexus of sensory nerves immediately beneath the vaginal epithelium. In addition, subpopulations of free nerve endings are sensitive to stretch, light mechanical touch, temperature, and pain (Hoyt, 2006).

### Afferent neural pathways from the genitalia

The ratio of small to large axons seen in nerves originating from the dermis of the human glans penis show an overwhelming majority of axons of small diameter (A6- or C-fibers) and scant larger myelinated fibers (>2 µm in diameter) (Halata & Munger, 1986).

At this point, it is relevant to mention a line of work done on sensual touch applied to hairy skin, in regions other than the genitalia. Some C-fibers are exquisitely sensitive to light (sensual) touch (Vallbo, Olausson, & Wessberg, 1999), but that has been shown only for hairy skin, i.e. not for the glabrous skin of the penis or for the clitoral and vaginal mucosae. In addition, human tactile C (CT) afferent pathways ultimately target the insular cortex (Björnsdotter, Löken, Olausson, Vallbo, & Wessberg, 2009; Björnsdotter, Morrison, & Olausson, 2010; Olausson et al., 2002, 2008). It is important to note that there is now fairly compelling evidence that the posterior insular cortex mediates the perception of pleasant caress (Morrison, Björnsdotter, & Olausson, 2011). There may even exist a somatotopic organization of the posterior insular cortex, with gentle touch of forearm evoking activation anterior to the one recorded in response to gentle touch of thigh. Interestingly, it appears that the two systems are partially antagonistic as CT stimulation in a patient lacking large myelinated afferents induced activation of posterior insular cortex while evoking deactivation in somatosensory cortex (Olausson et al., 2008). This potential antagonism could be helpful to understand why meta-analyses of brain responses to visual sexual stimuli show activation in SII, but not in SI.

### A hypothesis regarding the organization of genital somatosensory pathways

In connection with the widely different sensations evoked in the various reviewed studies, it may be useful to recall the classic distinction between two categories of somatosensory systems: epicritic and protopathic (Kandel, Schwartz, & Jessell, 2000). In response to stimuli, the protopathic system gives rise to affectively-laden sensations, either unpleasant or pleasant. By contrast, it carries little information about the localization and fine attributes of the stimulating agent. Conversely, the epicritic system mediates the ability to detect the localization of an object on the skin, to recognize the shape of an object being held, to achieve two-point discrimination, i.e. the spacing of two points being touched simultaneously (Semmes, 1969). Modern terminology favors the distinction between ‘fine touch’ (or discriminative touch) and ‘crude touch’. The posterior column-medial lemniscus pathway is responsible for sending fine touch information to SI via the medulla and the thalamus. Crude touch fibers are carried by the anterior spinothalamic tract and convey signals to the cingulate cortex, the SI, and the insular cortex.

Could it be that epicritic, fine touch, sensations and protopathic, crude touch, ones correspond to the distinct localizations on SI mentioned above? In that case, the study by Kell et al. (2005) would suggest that epicritic sensations correspond to the lateral postcentral gyrus. Conversely, those studies where visually-induced erection was associated with activation in the paracentral lobule (Moulier et al., 2006; Mouras et al., 2008) would suggest that protopathic sensation is related to activation of this medial region. Similarly, the study where manual stimulation of the clitoris was associated with activation in the paracentral lobule (Komisaruk et al., 2011) would suggest that clitoral protopathic sensation is related to activation of that medial region. However, this interpretation (epicritic sensation mapped onto lateral SI vs. protopathic sensation mapped onto medial SI) is not consistent with reports of only lateral activation associated with visually-induced erection (Hu et al., 2008; Redouté et al., 2000).

With regard to the above interpretation, studies based on electrical stimulation of the clitoris or the penis should result in activation of both localizations as electrical stimulation is likely to stimulate every kind of sensory fibers and hence to induce both kinds of sensations. Actually, apart from the study by Bradley et al. (1998), these studies reported activation in either one or the other localization (mesial: Allison et al., 1996; Guerit & Opsomer, 1991; Haldeman et al., 1983; Nakagawa et al., 1998; Yang & Kromm, 2004; lateral: Michels et al., 2010). However, it should be emphasized that most of these studies surmised rather than demonstrated the localizations of cortical activation.

In keeping with the above-proposed interpretation, we propose that part of the sensory afferents from the genitalia travel up to the primary sensory cortex, via the posterior column-medial lemniscus pathway (posterior column of spinal cord, then medial lemniscus, then ventral posterolateral nucleus of thalamus). This pathway conveys fine touch. The other part of the genital sensory afferents ascends to the cortex, via the anterior spinothalamic tract pathway. The first neurons of this pathway form the spinothalamic tract and synapse with secondary neurons in the posterior horn of the spinal cord. These secondary neurons ultimately synapse with third neurons in several nuclei of the thalamus – including the medial dorsal, ventral posterior lateral, and ventral medial posterior nuclei. From there, signals go to the cingulate cortex, the SI, and insular cortex, respectively (Behrens et al., 2003; Craig, 2003; Foreman, 1999; Vogt, Sikes, & Vogt, 1993). Inputs from the ventral medial posterior nucleus are highly specialized to convey homeostatic information such as pain, temperature, itch, local oxygen status, and sensual touch. Overall, given the evidence on the role of the posterior insular cortex in processing sensual touch from hairy skin and on processing afferent information regarding penile erection (Moulier et al., 2006; Mouras et al., 2008), we hypothesize that genital pleasurable sensations are represented in the posterior insula.

Furthermore, there are connections between the areas mentioned above. In monkeys, SII is densely interconnected with SI (Disbrow, Litinas, Recanzone, Padberg, & Krubitzer, 2003). In addition, the posterior insula connects reciprocally with SII. It is likely that SII sends information to the posterior insula so that genital sensations become part of sensual feelings and of the emotional component of sexual arousal (Jordan, Fromberger, Stolpmann, & Müller, 2011; Stoléru et al., 2012).

## Conclusion and recommendations

The findings of the present study tentatively support the interpretation that there are two distinct representations of genital sensations in SI, one on the medial surface and the other on the lateral surface. Further studies are needed to determine whether the discrepancy between studies regarding the localization of penile representation could be explained by the possibility that two kinds of penile sensations could be represented in different cortical locations, i.e. one on the lateral surface related to superficial skin stimulation and the other related to the perception of tumescence proper that would be located on the medial surface.

The four meta-analyses of brain responses to visual sexual stimulation have reported an activation in SII and the posterior insula.

Many studies have investigated the somatotopic organization of genitalia without considering the unique character of sensations originating in this region. In view of the erogenous character of sensations originating in the genitalia, future studies on this topic should systematically assess qualitatively as well as quantitatively the erotic, i.e. sexually stimulating and/or sexually pleasurable – characteristics of sensations felt by subjects in response to stimuli. In order to better understand the somatosensory representation of genitalia, it may be useful to combine in the same study both kind of tactile stimulation, i.e. inducing or not inducing pleasurable sensations. In males, for instance, one way to do this could involve stimulating the same area of the penis with an identical tactile stimulus both when the penis is flaccid and when it is erected. Another issue is related to the specific characteristics of the sensitivity of the glans penis and possibly of the clitoral glans. The glans penis has been suggested to be endowed with protopathic sensitivity only, which would justify to stimulate separately the glans and the shaft of the penis or clitoris in subsequent studies. More generally, the various parts of the genitalia (glans penis, penile shaft, scrotum, clitoris, labia minora, labia majora) are the sources of distinct sensations so that their neural correlates should not be studied as if genitalia were one homogeneous area.
